# Simultaneous bilateral distal biceps tendon ruptures repaired using an endobutton technique: a case report

**DOI:** 10.1186/1752-1947-7-213

**Published:** 2013-08-23

**Authors:** Mark P DaCambra, Richard EA Walker, Kevin A Hildebrand

**Affiliations:** 1 Division of Orthopaedic Surgery, Medical Education, Royal Columbian Hospital, University of British Columbia, 330 East Columbia Street, New Westminster, BC V3L 3W7, Canada; 2 Department of Radiology, Faculty of Medicine, University of Calgary, Room 812, North Tower, Foothills Medical Centre, 1403-29th Street NW, Calgary, AB T2N 2T9, Canada; 3 Section of Orthopaedic Surgery, Health Sciences Centre, University of Calgary, 3330 Hospital Drive NW, Calgary, AB T2N 4N1, Canada

## Abstract

**Introduction:**

The simultaneous rupture of both distal biceps tendons is a rare clinical entity that is difficult to treat and can have poor outcomes. A variety of treatment and rehabilitation options exist and have been reported for single sided and staged bilateral repairs, but none have described an approach for acute bilateral ruptures. Repairing distal biceps tendon ruptures using a single anterior incision and a cortical suspensory button technique has become increasingly popular in recent years. We present a report of our surgical approach using an endobutton technique and rehabilitation algorithm for this unusual injury pattern.

**Case presentation:**

A 43-year-old Caucasian man presented with acute onset bilateral elbow pain while lifting a large sheet of drywall off the ground. He initially felt a ‘pop’ on the right and almost immediately felt another on the left after having to quickly shift the weight. He was unable to continue working and sought medical attention. His pain was predominantly in his bilateral antecubital fossae and he had significant swelling and ecchymoses. His clinical examination demonstrated no palpable tendon, a retracted biceps muscle belly, and clear supination weakness. Magnetic resonance imaging was performed and showed bilateral distal biceps tendon ruptures with retraction on both sides. After discussion with our patient, we decided that both sides would be repaired using a single anterior incision with endobutton fixation, first his right followed by his left six weeks later.

**Conclusion:**

Overall, our patient did very well and had returned to full manual work by our last follow-up at 30 months. Although he was never able to return to competitive recreational hockey and was left with mild lateral antebrachial cutaneous nerve dysesthesias on his right, he felt he was at 85% of his premorbid level of function. We describe what we believe to be, to the best of our knowledge, the first case of simultaneous bilateral distal biceps tendon ruptures successfully treated with a single-incision endobutton technique, which represents a valid option in managing this difficult problem.

## Introduction

Simultaneous distal biceps tendon rupture is a rare diagnosis and this injury can have a devastating effect on level of function. Unilateral ruptures are treated with one of multiple surgical options, with variations in surgical approach, suture type, and fixation method to bone and tendon. Currently, there is a paucity of literature on the most appropriate surgical technique, timing of surgery and rehabilitation protocol in the patient with bilateral concomitant ruptures. We found a total of 31 reported cases of bilateral distal biceps tendon rupture in the English language literature [1-6]. Of these, there are only four reported cases of simultaneous bilateral ruptures [1-3,5], and none were repaired in the acute setting. To the best of our knowledge, this study represents the first report of an acute presentation of simultaneous bilateral distal biceps tendon ruptures.

## Case presentation

A 43-year-old right-hand dominant Caucasian man presented to our emergency department with bilateral elbow pain. He was lifting a large sheet of drywall into position at chest level, when he felt a sudden sharp pain in the anterior aspect of his right elbow. He immediately compensated by shifting the weight to his left arm, incurring another eccentric contraction at his elbow, when he felt a similar pain on his left side. The pain and subsequent weakness in both elbows persisted precipitating his visit.

His medical history was significant only for episodes of right lateral epicondylitis in the past treated with corticosteroid injections and bracing. He had been asymptomatic from this for a number of years. He also had mild chronic low back pain and a remote tonsillectomy and adenoidectomy. He took no regular medications, had no allergies, had never smoked cigarettes and drank one to two beers per week. He had been working in manual labor for 15 years, and enjoyed golf, hockey and playing the guitar.

Physical examination of his right elbow demonstrated an obvious deformity in the contour of the biceps muscle and tendon distally. There was significant ecchymoses present in the antecubital fossa extending distally down his anteromedial forearm. There was clear supination weakness and no tendon was palpable. The range of motion on his right was 5/0/134 degrees of flexion-extension, pronation of 75 degrees and 82 degrees of supination.

His left elbow biceps muscle belly extended more distal than on his right and he did have 4/5 supination power. He was, however, quite tender to palpation in the region, and no obvious tendon was palpable. The range of motion on his left was 0/0/134 degrees, with 73 degrees of pronation and 85 degrees of supination.

Median, anterior interosseous, posterior interosseous, radial, ulnar and musculocutaneous nerve function was intact bilaterally, and vascular status was normal.

**Figure 1:**
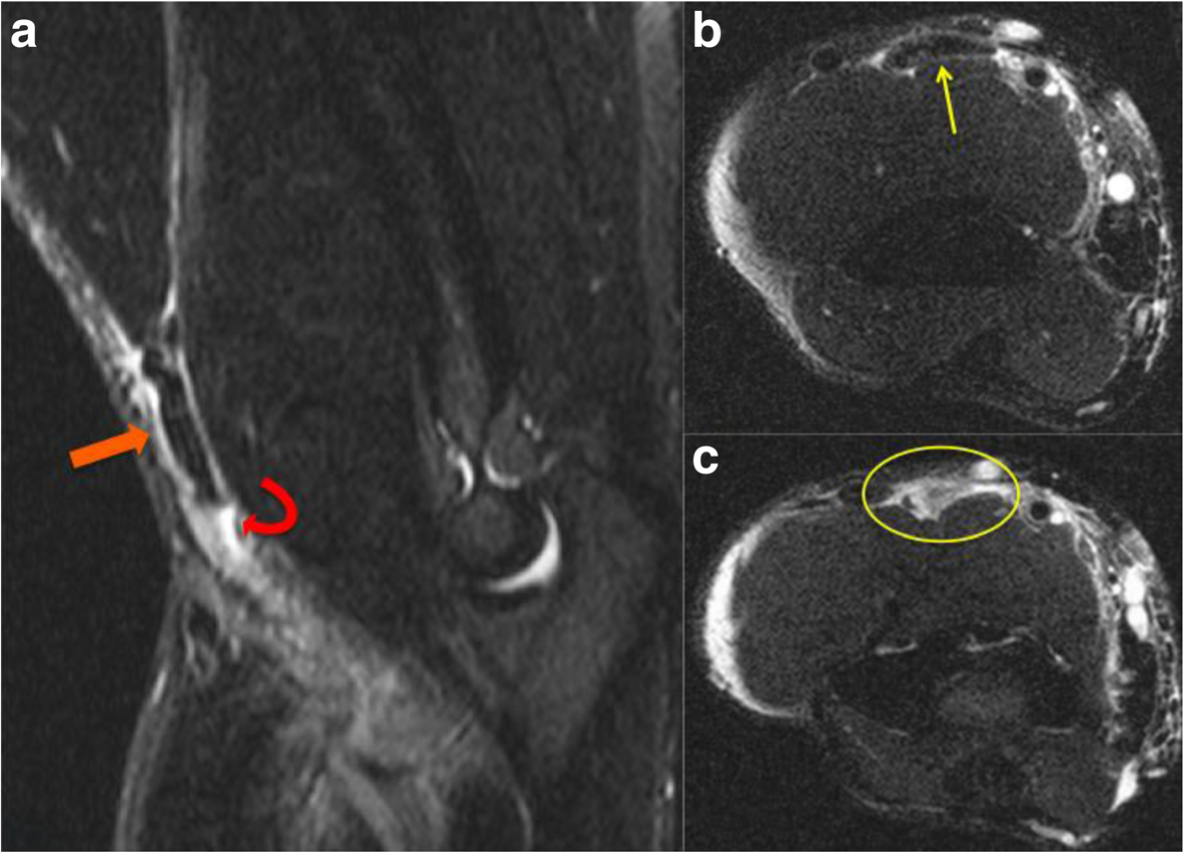
**Magnetic resonance imaging of the right elbow. (a)** Sagittal short tau inversion recovery image demonstrates a full thickness, complete tear of the distal biceps brachii tendon with proximal retraction (curved red arrow demarcates the retracted end of the torn tendon). Note the high signal intensity edema surrounding the thick, retracted tendon (thick orange arrow). **(b,c)** Axial fat-suppressed fast spin echo T2-weighted images demonstrate the thickened tendon superiorly (thin yellow arrow in **b**) with high signal fluid filling the defect left by the torn, retracted biceps brachii tendon more inferiorly (yellow oval in **c**).

**Figure 2:**
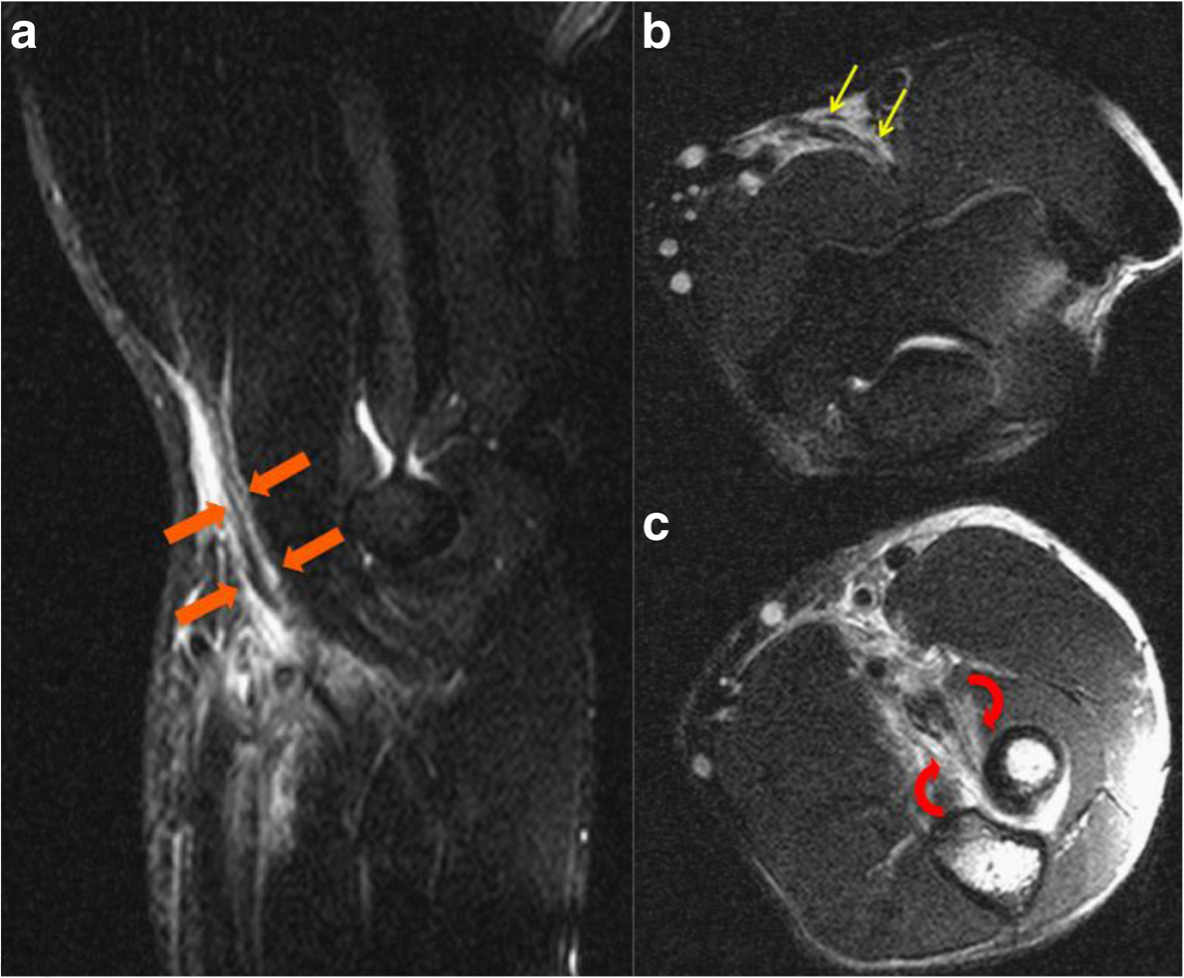
**Magnetic resonance imaging of the left elbow. (a)** Sagittal short tau inversion recovery image reveals high signal edema surrounding an attenuated distal biceps brachii tendon (thick orange arrows). **(b, c)** Axial fat-suppressed fast spin echo T2-weighted images demonstrate some linear high-signal intensity within the attenuated distal biceps brachii tendon consistent with longitudinal partial-thickness tearing (thin yellow arrows in **b**). At the level of the radial tuberosity, there is a full thickness tear at the tendon insertion (curved red arrows in **c**) with 1cm to 2cm of tendon retraction.

Magnetic resonance imaging was subsequently obtained showing complete tears with 6.7cm of retraction on the right (Figure 1), and with 1cm to 2cm of retraction on the left (Figure 2).

After discussion with our radiology department, our patient, and his family, we decided to perform a staged distal biceps tendon repair first on his right and then six weeks later on his left.

**Figure 3:**
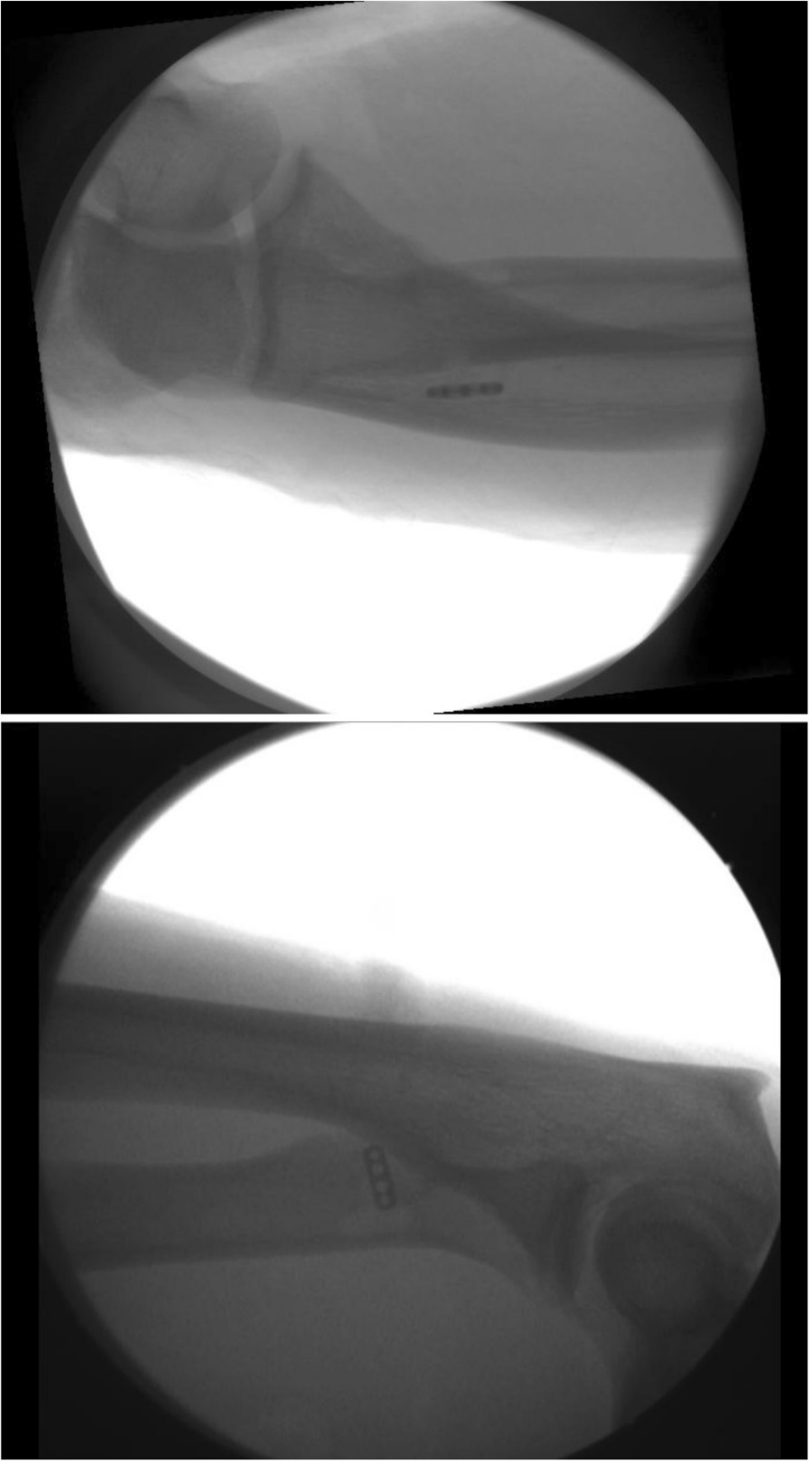
Anteroposterior and lateral intraoperative fluoroscopic image depicting the position of the suspensory cortical button after fixation.

The technique used for both sides was a single anterior incision approach with tendon fixation provided with a suspensory cortical button (Endobutton; Smith and Nephew Endoscopy, Andover, MA, USA) [7]. Briefly, a tourniquet was applied to his upper arm and a 5cm anterior curvilinear transverse incision was made, and carried distally on the medial border of his brachioradialis. The lateral antebrachial cutaneous nerve (LABCN) was identified and the retracted end of his biceps was completely detached and easily visualized. The vacant tendon tract leading down to the radial tuberosity was exploited, and with his elbow fully supinated and extended, the radial tuberosity was exposed. A bicortical hole was made first with a 4.5mm drill in the anatomic medial aspect of the biceps insertion on the radial tuberosity. A second and third unicortical 4.5mm drill hole was made just proximal and distal to the first. A high speed burr was used to make the unicortical opening sufficiently wide to accept the tendon end. The tendon end was debrided of interposing tissue, and two fiberwire (Arthrex Inc., Naples, FL, USA) sutures were used to secure the tendon to the two central holes of the endobutton. Bunnell-type sutures were placed in the medial and lateral margins of the tendon with the knots placed proximally. Sutures were then placed in the leading and trailing holes of the endobutton and threaded through a long straight-eyed needle. The needle was advanced through the bicortical hole dorsally, with tension applied to the lead suture in order to pass the endobutton and deliver the tendon end into the tunneled insertion site. The endobutton was flipped securing the repair. The strength of the repair was confirmed by lifting his arm off the table by the biceps tendon using a retractor. Intra-operative fluoroscopic images were obtained confirming that the button was on the dorsal side (Figure 3).

The same postoperative protocol was used for each elbow. In the operating room, a plaster splint was fashioned with the elbow at 90 degrees flexion, the forearm in neutral rotation and the wrist in neutral. All digits were free. One week after the operation, a removable splint with the extremity in the same position was made and physiotherapy started. This consisted of passive elbow flexion and active extension exercises with his forearm supinated. Elbow flexion was full while a 30 degree extension block was observed. Passive full supination, and active pronation limited to 50 degrees with the elbow at 90 degrees flexion was also performed. This was implemented for five weeks. At six weeks, the removable splint was stopped, and strengthening and stretching exercises were added to obtain full motion. Resistance was progressed to a maximum of 4.54kg (10 pounds). Three months after the operation, all restrictions were removed.

Initially, our patient experienced postoperative paresthesias in the anterolateral aspect of his right forearm. This gradually improved but had not completely resolved when he resumed full work activities four months after the left-sided repair. The rest of his neurovascular examination was normal.

Our last follow-up was approximately 30 months after the second operation. At that point, our patient was still working full-time without major difficulties. He felt he was at 85% of his premorbid function. On his right, he had 6/0/128 degrees of flexion-extension, 75 degrees of pronation and 85 degrees of supination. On his left, he had 6/0/145 degrees of flexion-extension, 80 degrees of pronation and 89 degrees of supination. He still had a small area of numbness over his anterolateral right forearm and said he had not been able to return to hockey or golf. Disabilities of the Arm, Shoulder and Hand (DASH) scores were obtained at the time of initial evaluation and 30 months after surgery. Pre-injury DASH scores were based on our patient’s recollection of premorbid arm function. The DASH scores went from 41 before the operation to 20 on his left and 29 on his right 30 months later (Additional file 1).

## Discussion

Simultaneous distal biceps tendon rupture is a rare diagnosis and the most appropriate management of this injury depends on numerous factors. Bayat *et al.* reported the case of a 50-year-old rock climber sustaining bilateral injuries while climbing, who presented for surgical treatment two years after the injury [2]. He went on to have tendon reconstructions with ipsilateral fascia lata grafts on the right and left, staged six months apart. Rokito and Iofin reported on a 51-year-old recreational weightlifter getting injured while doing rapid preacher curls with 40.82kg (90 pound) barbells [3]. He presented approximately six weeks post injury and underwent primary repair on one side at seven weeks post injury, followed by allograft reconstruction on the other side 13 weeks later. Visuri and Lindholm presented the case of a young bodybuilder who ingested megadoses of anabolic androgenic steroids for six years who presented late with bilateral ruptures [1]. The final case comes from the retrospective review of Bell *et al.*, who reported on 26 cases, one of which included a simultaneous bilateral rupture [5]. The timing of the surgery and type of surgery done on this individual were not reported. Because of the acute presentation in our case, we were able to perform the second surgery six weeks after the injury, enabling us to perform a primary repair without issue.

Bilateral injuries confound the treatment of distal biceps tendon rupture considerably. The major questions to be answered are whether the injuries occurred contemporaneously or at different times, and if they are acute (less than six weeks) or chronic. The definition of six weeks as acute is arbitrary but provides a general guideline after which primary repair becomes increasingly difficult. If the injuries happened in the same incident and are acute, bilateral repair is an option. If both are chronic in this setting, bilateral reconstruction is warranted. If the injuries did not occur at the same time, three possibilities exist: both are acute, that is distinct, injuries but both within six weeks; one injury is acute and one is chronic; or both are chronic. Based on this is the determination of whether repair or reconstruction of one or both sides can be made (Additional file 2).

Regardless of whether a repair or reconstruction is warranted, the decision to repair both at the same time or in a staged manner must be made. Numerous factors must be considered such as the patients’ social situation, hand dominance, occupation, medical comorbidities and general health. There is currently no evidence to guide this, and ultimately the decision is made in collaboration with the patient, their family and the heath care team.

The most appropriate surgical technique for distal biceps tendon repair in general is a source of debate in the literature. Unlike our study using the single-incision approach with the endobutton [7], the technique of primary repair used in all of the above cases was the two-incision modified Boyd-Anderson approach [8], with fixation using either suture through bone tunnels or suture anchors. The most common complication using an anterior approach is LABCN and superficial radial nerve palsy, the latter of which we did see in our case. The incidence of LABCN palsy ranges from 5% to 44% in the literature, and seems to depend on the size of the anterior exposure [9-12]. The two-incision approach introduced by Boyd and Anderson was adopted because of the high rate of anterior nerve palsies seen with an extensive anterior approach. The posterior dissection, however, resulted in proximal radioulnar synostosis and heterotopic ossification in a number of cases [13]. Since the advent of better fixation methods resulting in smaller dissections, a smaller one-incision anterior approach appears to have a similar side effect profile. Ultimately, the decision is made in collaboration with the patient and is based on surgeon experience and patient preference because the functional outcomes between the two approaches appear similar.

With regards to fixations methods, the endobutton technique using an anterior approach described by Bain *et al.*[7] has been shown to have the highest peak load to failure and cyclical load to failure in multiple biomechanical studies comparing various fixation techniques [14]. It also may have one of the highest degrees of tendon displacement post cyclical loading, although the clinical significance of this is unclear. There is currently no study to date outlining the complication profile utilizing the endobutton in a single-incision technique.

## Conclusions

Overall, our patient has done reasonably well with this rare injury pattern, and he feels he is at 85% of his premorbid function. He has good strength and is able to participate fully in his work. His range of motion is excellent and he has no synostosis or clinical manifestations of heterotopic ossification. He has however, a persistent LABCN paresthesia and occasional mild pain and stiffness, which he feels does interfere with his level of function to an extent as reflected in his DASH scores. Clearly, more research is needed to guide treatment and improve outcomes of this injury.

## Consent

Written informed consent was obtained from the patient for publication of this case report and accompanying images. A copy of the written consent is available for review by the Editor-in-Chief of this journal.

## Abbreviations

DASH: Disabilities of the arm shoulder and hand; LABCN: Lateral antebrachial cutaneous nerve.

## Competing interests

The authors declare that they have no competing interests.

## Authors’ contributions

KH identified the patient, performed the surgeries, followed the patient, and was a contributor to the writing of the paper. RW assessed and reported on the diagnostic imaging involved in the case and was a contributor to the writing of the paper. MD assisted in the surgeries, interviewed the patient, and was a major contributor to the writing of the paper. All authors read and approved the final manuscript.

## Supplementary Material

Additional file 1 DASH scores reflecting pre-injury, preoperative, and 30 months postoperative arm function. Click here for file

Additional file 2 **Treatment algorithm for bilateral distal biceps tendon ruptures.** * Patient factors include social support, hand dominance, occupation, medical comorbidities and general health. Click here for file
