# Supplementary figures and images for: Simultaneous bilateral distal biceps tendon ruptures repaired using an endobutton technique: a case report
Source: J Med Case Rep. 2013 Aug 23;7:213. doi: 10.1186/1752-1947-7-213 (PMC3766066; doi:10.1186/1752-1947-7-213)

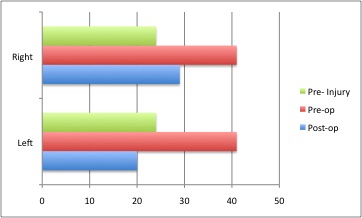


.

Supplement: Additional file 1 — DASH scores reflecting pre-injury, preoperative, and 30 months postoperative arm function. [file 1752-1947-7-213-S1.docx]

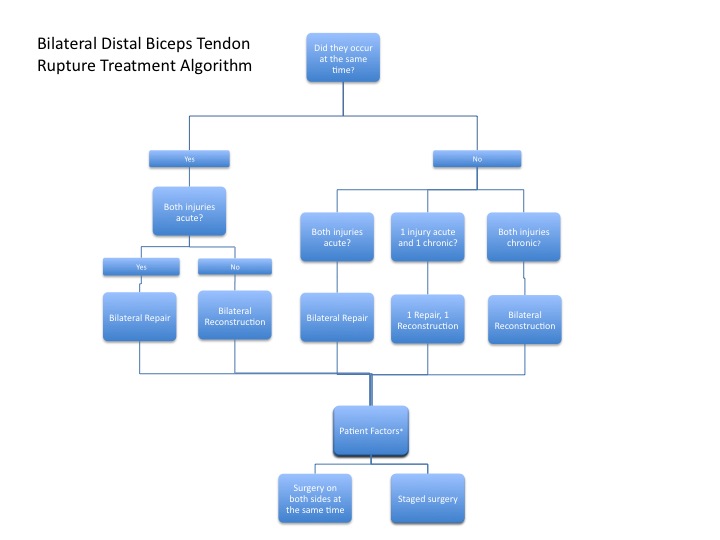

Supplement: Additional file 2 — Treatment algorithm for bilateral distal biceps tendon ruptures. * Patient factors include social support, hand dominance, occupation, medical comorbidities and general health. [file 1752-1947-7-213-S2.jpeg]
